# Automated detection of glaucoma using structural and non structural features

**DOI:** 10.1186/s40064-016-3175-4

**Published:** 2016-09-09

**Authors:** Anum A. Salam, Tehmina Khalil, M. Usman Akram, Amina Jameel, Imran Basit

**Affiliations:** 1 National University of Sciences and Technology, Islamabad, Pakistan; 2 Bahria University, Islamabad, Pakistan; 3 Armed Forces Institute of Ophthalmology, Rawalpindi, Pakistan

**Keywords:** Computer aided diagnostics, Cup to disc ratio, Fundoscopy, Glaucoma detection, Machine learning

## Abstract

Glaucoma is a chronic disease often called “silent thief of sight” as it has no symptoms and if not detected at an early stage it may cause permanent blindness. Glaucoma progression precedes some structural changes in the retina which aid ophthalmologists to detect glaucoma at an early stage and stop its progression. Fundoscopy is among one of the biomedical imaging techniques to analyze the internal structure of retina. Our proposed technique provides a novel algorithm to detect glaucoma from digital fundus image using a hybrid feature set. This paper proposes a novel combination of structural (cup to disc ratio) and non-structural (texture and intensity) features to improve the accuracy of automated diagnosis of glaucoma. The proposed method introduces a suspect class in automated diagnosis in case of any conflict in decision from structural and non-structural features. The evaluation of proposed algorithm is performed using a local database containing fundus images from 100 patients. This system is designed to refer glaucoma cases from rural areas to specialists and the motivation behind introducing suspect class is to ensure high sensitivity of proposed system. The average sensitivity and specificity of proposed system are 100 and 87 % respectively.

## Background

Glaucoma is a chronic ocular disorder which can cause blindness if left undetected at an early stage. The World Health Organization has declared Glaucoma to be the second largest cause of blindness all over the world and it encompasses 15 % of the blindness cases in the world which makes 5.2 million of the world’s population (Thylefors and Negrel 1994) and the number is expected to increase up to 80 million by 2020 (Quigley and Broman 2006). Glaucoma originates from increase in intraocular pressure caused by aqueous humor, a fluid produced by the eye. In normal eye, a balance is maintained as the amount of liquid produced is equal to the amount of liquid discharged by the eye. However, in glaucoma the liquid do not flow out of the eye and increases stress on the eye resulting in the damage of optic nerve which is responsible for brain and eye communication. Increase in pressure with time results in severe destruction to optic nerve and may ends up with irreversible blindness (http://www.geteyesmart.org/eyesmart/diseases/glaucoma/). Increase in intraocular pressure (IOP) causes no early symptoms or pain which makes the early detection of glaucoma complex and thus often called “Silent thief of sight”.

Structural changes that occur in the internal eye are one of the vital sources to detect glaucoma. Fundoscopy and Optical Coherence Tomography (OCT) are two modern biomedical imaging techniques that enable ophthalmologists in examining the inner details of eye to detect abnormalities. In Fundoscopy (The Basics: Direct and Indirect Ophthalmoscopy 2015), also known as Ophthalmoscopy, subject’s internal eye is enlightened using a light beam reflected from the mirror mount in the device. A piercing in the mirror center helps the observer to view the enlightened region. Light rays reflected from the subject’s eye are converged at the observer’s retina forming a vision.

**Fig. 1:**
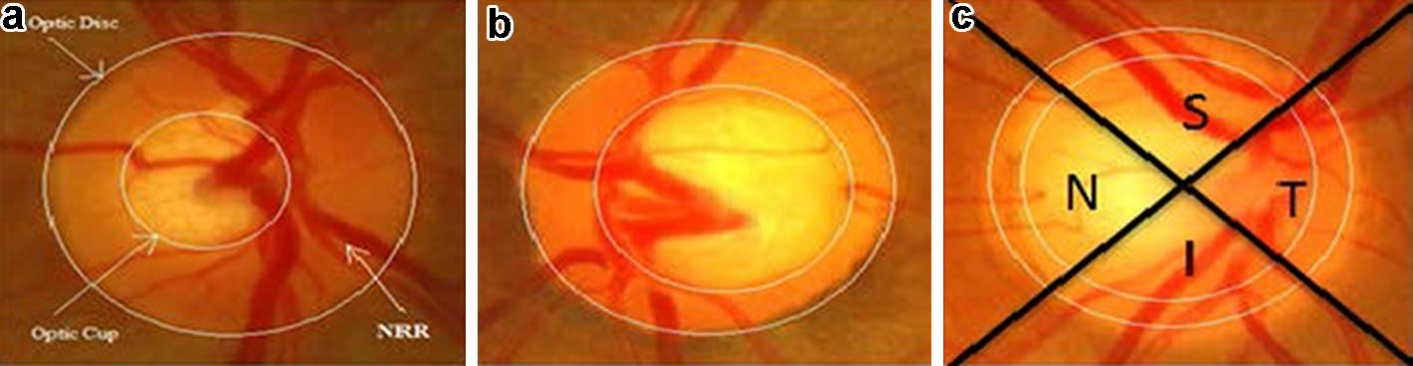
**a** Normal eye; **b** eye with moderate glaucoma; **c** eye with severe glaucoma

Fundoscopy enables ophthalmologists to examine the Optic Disc. Optic disc appears as a yellowish circular body, centered with optic cup which is slightly brighter area than Optic disc. Figure 1 shows a normal eye fundus image. Circular rim area between optic cup and optic disc is called neuroretinal rim (NRR). Ratio of Cup area to disc area called Cup to Disc Ratio (CDR) is one of the noticeable structural change that occurs if glaucoma progress. CDR value ≤0.5 indicates normal eye (Murthi and Madheswaran 2012). Cup size increases in glaucomatous eyes, resulting in increase of CDR and decrease in NRR. Thus, CDR and NRR are two key structural changes to detect glaucoma using Fundoscopy. Figure 1a shows a normal fundus eye image with normal CDR i.e. ≤0.5, whereas Fig. 1b, c shows a glaucoma eye fundus image. Along with the change in CDR, Fig. 1b, c depicts the change in color intensity values of glaucoma versus non-glaucoma images. In glaucoma image, due to increase in the size of cup brighter region in the optic disc increases thus increasing the overall image entropy, mean, variance and color spatial and textural information. Thus along with CDR and NRR, image intensity and textural information can also be used to detect glaucoma.

NRR thickness based on Inferior, Superior, Nasal and Temporal (ISNT) rule is also being used as a reference for glaucoma detection. Figure 1c shows the labeled ISNT regions in the rim area of fundus image for right eye. Inferior region is the bottom region of NRR, Superior is the top region of NRR, Nasal and Temporal are the right and left regions of NRR in case of right eye, in case of left eye nasal and temporal are the left and right regions respectively. ISNT rule states that in a normal eye thickness of NRR is such that inferior region > superior region > nasal region > temporal region (Ruengkitpinyo et al. 2015). Rest of the paper is organized as follows. Section “Literature review” is comprised of the recent relayed work, followed by “Proposed methodology” section composed of a detailed explanation of the proposed methodology. Section “Results ” comprised of the results and a quantitative analysis on the results obtained from the algorithm. Section “Discussion and conclusion” concludes the paper.

## Literature review

An accurate Glaucoma detection is among one of the crucial requirements to control its progression and reduce the risk of blindness. In biomedical imaging glaucoma detection is one of the active researches being done in the field of autonomous glaucoma detection systems to provide state of art computer aided design (CAD) tool that can aid ophthalmologists in early glaucoma detection. Many image processing, computer vision and machine learning techniques and tools are being used to excel in this research field and come up with more accurate results that might help in more accurate and early glaucoma diagnosis. Some state of art methodologies are being discussed in the upcoming section.

An Optic disc localization algorithm (GeethaRamani and Dhanapackiam 2014) implemented template matching technique to locate optic disc center. Template was created by averaging the images in the database. Green plane was processed further using some morphological operations to detect the Optic disc. In 2014, a disc localization algorithm (Akhade et al. 2014) performed principal component analysis (PCA). Most significant principal component was further processed to remove vessels using morphological operations. Circular Hough transformation was applied to detect the circular body from the resultant fundus image. Tan et al. (2010) used Gaussian Mixture Models (GMM) to extract cup region from fundus images. Resulting cup region has good boundary results in temporal region, while cup extraction algorithm in ARGALI performs well in nasal region, thus a hybrid cup extraction was done by fusion of both ARGALI detected boundary and proposed algorithm detected boundary. Algorithm was tested on 71 images and 14 % error reduction was observed. Cup detection algorithm based on vessel kinking was proposed in Damon et al. (2012). Algorithm proceeds with detecting vessels by classifying some patches of interest by using features like mean and standard deviation of a fused image formed after computing wavelets of edges of green red component and gradient of green component. Vessel kink detection was completed by localizing maximum curvature of the detected vessels. Algorithm evaluation was done on 67 images, and has reduced 43.3 % errors in cup boundary detection. A spatial heuristics based analysis was done to extract cup (Wong et al. 2012) after fusing two segmentation techniques i.e. Level set technique applied on green channel to extract brightest pixels from the image and Color histogram approach which selects pixels in a certain range in red, green and blue plane by region growing. Cup boundary was extracted by fusing the two segmentation techniques for the reason that both techniques perform differently in the inferior, superior, nasal and temporal regions. Best key point at each phase shift was determined using Bayesian score to select the boundary of cup from the two segmentation techniques. Local patching i.e. Super pixel segmentation and grid decomposition techniques (Xu et al. 2014) were applied to segment cup from the rest of the fundus image.

Autonomous Glaucoma detection algorithm was proposed in Dutta et al. (2014) based on cup and disc region segmentation. Double thresholding was done to segment background from ROI. Hough transform was used to estimate cup and disc boundary. Computer aided glaucoma detection system was proposed (Ahmad et al. 2014; Khan et al. 2013) that analyzes a fundus image using CDR and ISNT rule to classify as glaucoma or healthy. Algorithm preprocesses fundus images by cropping the image followed by Green plane extraction from RGB domain to detect cup and Value plane from HSV domain to detect optic disc. Binary image is obtained after thresholding followed by noise and vessel interference removal using morphological operations. Decision of glaucoma or healthy eye was done on basis of CDR and ISNT rule. 97 % accuracy was achieved. Cup to disc ratio of fundus images was computed using a segmentation technique (Poshtyar et al. 2013) to detect cup and disc. Prior to image segmentation, image was preprocessed and a rough estimate of optic cup center and disc center are made, followed by segmentation of that region by setting the average of gray level pixels as a threshold. CDR was computed and images were classified as healthy and glaucoma with success rate of 92 %. A Multi thresholding approach was proposed in Noor et al. (2013) to segment cup and disc from fundus image. Image was preprocessed followed by calculation of threshold value iteratively until reached a step where threshold reached a constant value. Image was segmented using this threshold to detect cup and optic disc separately. Yin et al. (2012) proposed a glaucoma detection system using CDR computation. Fundus image in RGB domain was used for disc extraction (Red plane) and cup extraction (Green plane). In optic disc detection Red plane is utilized and circular Hough transform is applied to detect the boundary pixels of optic disc. Optic cup extraction is done by processing green plane. Active shape modeling is done to detect cup boundary from the interpolated image. A cup and disc enhancement technique (Murthi and Madheswaran 2012) to improve CDR computation and increase the accuracy of calculated CDR was proposed which enhances the cup and disc detected by ARGALI by applying ellipse fitting to the boundaries detected. An algorithm for optic cup and disc detection was proposed in Kavitha et al. (2010) that processed red plane for disc detection and green plane for cup detection. Morphological operations were performed to remove vessels and unwanted noise. Manual threshold, component analysis and ROI based segmentation techniques were compared for cup and disc detection. A super pixels based glaucoma screening algorithm (Cheng et al. 2013) using cup to disc ratio was proposed in 2013. Optic cup and optic disc segmentation is done by dividing the fundus image to super pixels and extracting features for each super pixel region. Features were used to discriminate cup region or disc region. After feature extraction each region is passed to SVM, which classifies the super pixel as disc/non-disc or cup/non-cup region. Sparse dissimilarity coding algorithm (Cheng et al. 2015) was proposed from glaucoma screening using CDR using fundus images. Proposed methodology segments the optic disc by combining results from three disc segmentation techniques. First technique used Circular Hough transform followed by Active contour models, second technique used super pixel based feature extraction and classification, third technique used ellipse fitting. Best result of segmented disc from the three techniques was used for further processing.

Glaucoma progression occurs due to increase in IOP, resulting in an increased cup size. Cup is the brightest central region and thus increase in cup size results in change of intensity based and texture based properties of the fundus image. Research is being done in using state of art machine learning techniques (texture based and intensity based features) to classify images as glaucoma or non-glaucoma. High order spectral and textural features were used (Acharya et al. 2011) to detect glaucoma from fundus images. Fundus image is preprocessed followed by texture feature extraction and High order spectral features. Support vector machine (SVM), Naïve Bayes, Random Forest and sequential minimal optimization (SMO) were used to classify the images as glaucoma and non-glaucoma. Algorithm was found to be 91 % accurate with random forest classifier. A CAD algorithm for glaucoma using Haralick features was proposed in Simonthomas et al. (2014). GLCM of input fundus is computed for the four directions (0°, 45°, 90°, and 135°) and combined by summing up and averaging to get a single matrix composed from the four direction matrices. Haralick texture features of the resultant GLCM were computed and K nearest neighbor (KNN) classifier was used to classify the images as healthy or glaucomatous. Accuracy for a local data set comprised of 60 images was found to be 98 %. A review on machine learning techniques for autonomous glaucoma detection (Khalil et al. 2014) provided a deep analysis on different machine learning, feature selection, training techniques. A comparison between different spatial and frequency based features was made using different classifiers for training and testing. It was concluded that all the machine learning techniques using combination of features have ability to detect 85 % glaucoma cases. An algorithm (Raja and Gangatharan 2015) used Wavelet transformation based features to discriminate glaucoma from healthy eyes. SVM classifier using radial basis function kernel was used. tenfold cross validation was done to evaluate the algorithm’s accuracy.

All the proposed methodologies for autonomous glaucoma detection have used either structural features (CDR, RDR, and ISNT) or intensity based features individually. Glaucoma classification based on structural changes sometimes leads to in accurate results for the reason of inaccurate cup or disc segmentation due to the presence of bright lesions. Similarly, autonomous glaucoma detection using intensity based features or textural changes can mislead for the reason of non-uniform illumination or bright fringes introduced at the time of fundus image acquisition. Proposed methodology provides an algorithm for autonomous glaucoma detection using a fusion of results from CDR and feature based classification and also introduces a suspect class to cater the boundary cases. Cup and disc detection is done followed by an analysis of input fundus image based on CDR value, a decision based on CDR value is made after comparing it with the threshold i.e. 0.5. Results from CDR module of the proposed system are correlated with the results from machine learning module implemented using state of art machine learning techniques. Textural and intensity based features are computed from an image to construct a feature vector and classification is done using SVM. Final classification of an image is done after fusing the results from both modules to increase the sensitivity of the system.

## Proposed methodology

**Fig. 2:**
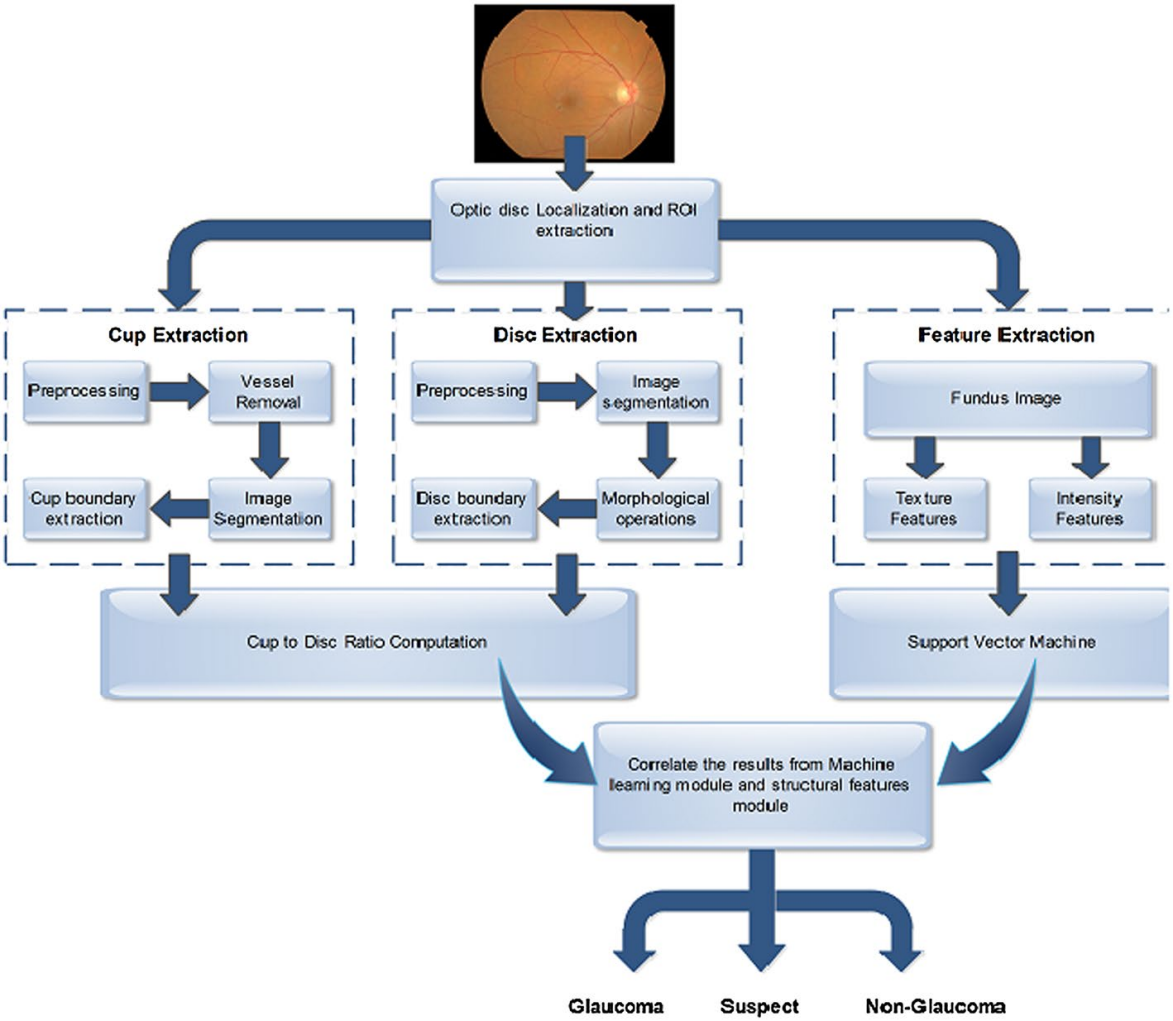
Proposed methodology

Glaucoma is an ocular condition whose progression leads to permanent blindness. Glaucoma is a chronic disease whose progression can only be stopped if detected accurately at an early stage. Proposed algorithm provides an automated glaucoma detection computer aided system that enables the ophthalmologists in early diagnosis of glaucoma patients with high accuracy. Algorithm takes a preprocessed fundus image and extracts optic cup and optic disc followed by CDR calculation. Intensity and textural features are extracted from the image to train and test the classifier. Result from glaucoma detection using CDR and features are combined to classify the image as glaucoma, non-glaucoma or suspect. Figure 2 depicts the complete methodology.

**Fig. 3:**
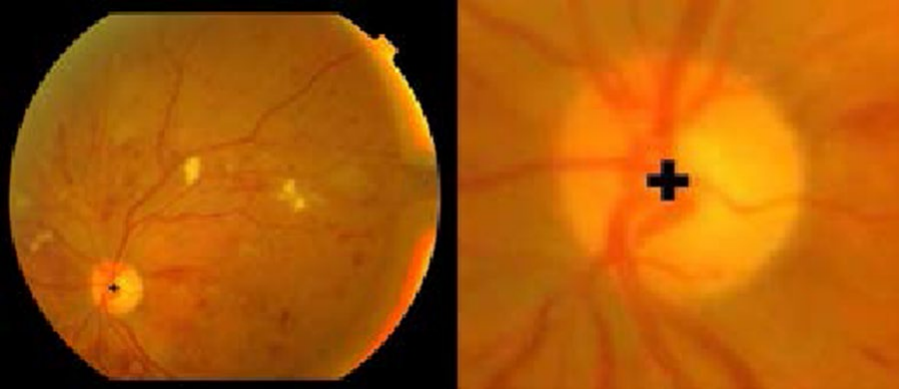
Optic disc localization and ROI extraction

Fundus image obtained is preprocessed before cup and disc detection to extract the Region of interest (ROI) i.e. the region containing optic disc known as optic disc localization (Salam et al. 2015). ROI extraction reduces the image size, thus reduces the time complexity of algorithm by processing only the required region. Moreover, fundus image might contain bright lesions or fringes which may be considered as optic disc. To remove the unwanted noise ROI is extracted from the fundus image to proceed further. Fundus image is preprocessed using contrast enhancement followed by applying Laplacian of Gaussian (LoG) (Usman et al. 2014) to detect blobs. Image is converted to binary image using a threshold value which selects 40 % of the bright pixels from the Gaussian kernelled image. All the detected blobs are the candidate regions for optic disc. Region with largest vessels density is finally selected as the optic disc location. Taking center of detected disc from candidate region, an area of 155 × 175 is cropped to extract ROI shown in Fig. 3.

### Cup detection

Optic cup is the central bright yellowish circular region in optic disc. Cup detection from fundus image is one of the challenging tasks as the color intensity of cup region does not differ much from the Optic disc region, which makes cup detection a complex task. Moreover, cup region is interfered with the blood vessels, as all the blood vessels originate from the optic nerve, located at the center of the optic disc. The vessels hide the cup region and small regions of cup are visible inside the optic disc.

#### Preprocessing

Fundus image is preprocessed to enhance the contrast between cup and disc. Contrast Stretching is an intensity normalization technique that enhances the contrast by stretching the intensity value range and map it on linear scale with Linear gamma correction γ = 1. Mapping the intensity values to a linear scale requires highest ($x_{hi}$) and lowest intensity ($x_{low}$) value of the range to be mapped from the input image and the desired highest ($y_{hi}$) and lowest intensity ($y_{low}$) value of the range of the resultant image. In (1) contrast stretching expression is shown.

1 $$y = (x - x_{low} )\frac{{y_{hi} - y_{low} }}{{x_{hi} - x_{low} }} + y_{low}$$

**Fig. 4:**
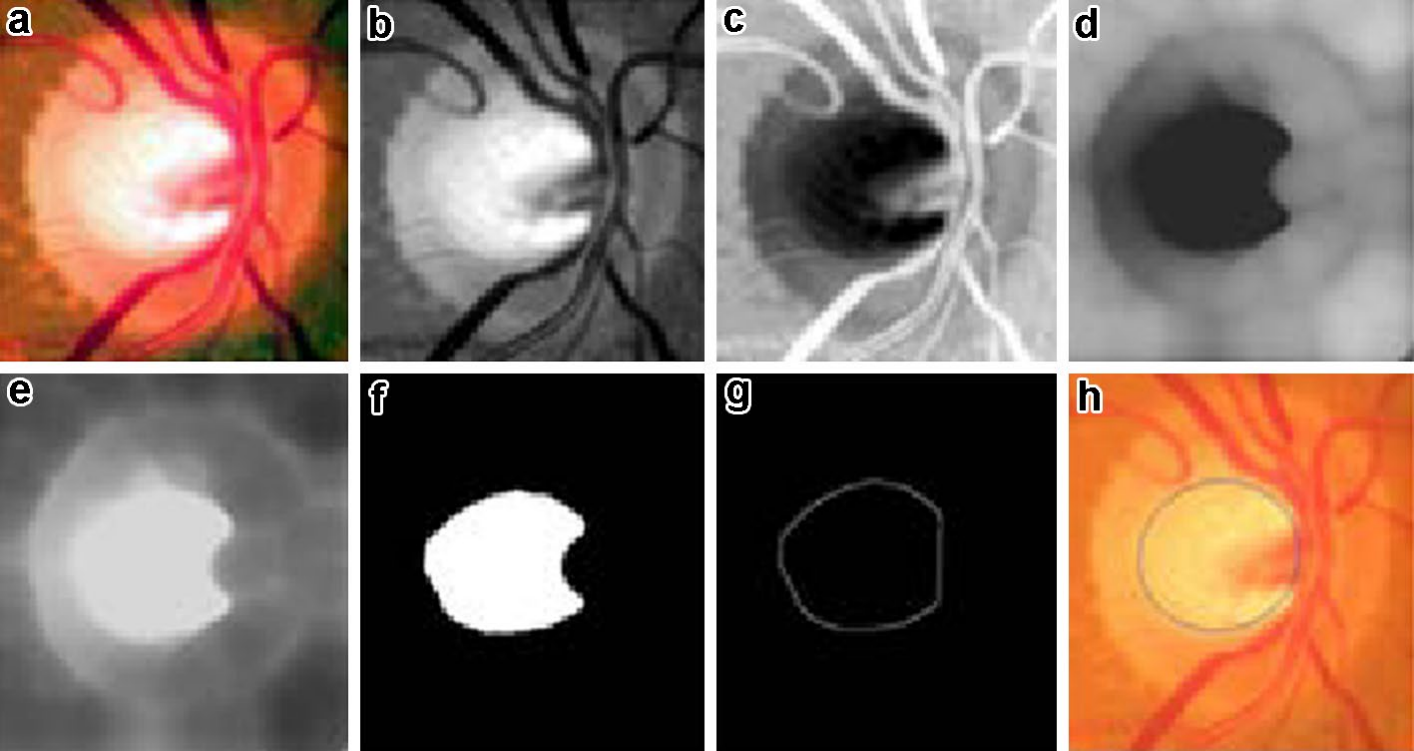
**a** Contrast enhanced RGB color space image; **b** extracted green plane; **c** negative transform; **d** vessel removal using opening; **e** negative transform; **f** region growing; **g** Edge extraction of convex hull from **f**; **h** detected cup after Ellipse fitting

Contrast stretching is applied on the Red, Green and Blue (RGB) plane separately to enhance the visual contents in the ROI of the three planes. Green plane contains the most significant information of optic cup, thus the resultant Green plane is processed further. Image is transformed to a negative using negative transform to get inverted intensity values. Negative transform of an L bit image with range of [0 2L] intensity values is obtained by subtracting each input intensity value x from highest intensity value. Figure 4a–c shows the results after contrast enhancement, Green plane extraction and negative transform respectively. Negative transform is represented in (2).

2 $$y = (2^{L} - 1) - x$$

#### Vessel removal

Optic cup region is interfered with many blood vessels as all the vessels originate from the center of the Optic disc, the region where Optic cup is located. Varying form image to image, some portion of cup is overlapped by the blood vessels. To detect cup from the resultant image (I), blood vessels impression should be lightened. Thus, gray level opening is performed using a ball shaped non-flat structuring element (S) of size radius 40 and height 40. Opening is a Morphological operation defined as erosion followed by dilation. Erosion will remove the unwanted objects i.e. blood vessels, followed by dilation to keep the other objects unaffected. Opening will remove all the blood vessels impression resulting in a smooth cup region as shown in Fig. 4d. Opening operation is represented in (3). In (3) Θ represents morphological erosion operation and ⊕ represents morphological dilation operation.

3 $$IoS = (I\varTheta S) \oplus S$$

#### Cup region extraction

Resultant image from the previous step is segmented using Region growing, a region based segmentation technique. Region growing is an iterative segmentation algorithm that takes an initial pixel as a seed point and grows the region based upon homogeneity of region using some criteria i.e. pixel intensity value, mean or variance. Iteration stops when certain defined criteria fails. At each step before a pixel in 4-neighbors is classified as a region pixel or non-region pixel using certain criteria region homogeneity is checked. In fundus image, cup region has different intensity values than rest of the region thus cup segmentation pixel intensity values and mean of region are measured for similarity and homogeneity. Iteration stops at the stage when intensity difference and mean of the new pixel increases from the given threshold i.e. 0.025. Assume I = [i (j, k)] for j = 1, 2, 3,…, M and k = 1, 2, 3,…, N is the image of size M × N having intensity value i(j, k) at jth row kth column, a region P(Z × Y) inside I is a connected region which grows after each iteration. Parameter to calculate homogeneity i.e. mean for the region S can be calculated as

4 $$\mu_{t,v} = \frac{1}{{\left( {Z \times Y} \right)}}\mathop \sum \limits_{v = - Y/2}^{v = Y/2} \mathop \sum \limits_{t = - Z/2}^{t = Z/2} i\left( {t,v} \right)$$

Pixel with maximum intensity value is selected as an initial seed point. At each step two conditions are checked before growing region

1. neighboring pixel N(x, y) from 4-neighbours should fulfill the intensity bound threshold condition i.e. P(m, n) − N(x, y) < intensity threshold
2. Including the pixel in the region should not disturb homogeneity i.e. Region homogeneity <threshold

Region growing segments the image such that the resultant image contains cup region and a background. Resultant image is shown in Fig. 4e. Resultant region may contain holes or unfilled portions, to fill the extracted cup region convex hull of the resultant image is obtained. Convex hull is the region of any object such that any line segments that join any two points of the object should lie within this region. Convex hull of any object is determined using four structuring elements Bi, i = 1, 2, 3, 4 in (5), (6) and (7).

5 $$X_{k}^{i} = \left( {X_{k - 1} *B^{i} } \right) \cup A$$

6 $$\left( {X_{k - 1} *B^{i} } \right) = \left( {X_{k - 1} \varTheta B^{i} } \right) \cap \left[ {X_{k - 1\Theta}^{c} \left( {{\text{W}} - B^{i} } \right)} \right]$$

7 $$D^{i} = X_{k}^{i} , C\left( A \right) = \mathop \coprod \limits_{i = 1}^{4} D^{i}$$

Let A be Image after region growing. Algorithm starts with $X_{0}^{i}$ = A, and iteration continues k = 1, 2, 3,… till no change appears in $X_{k}^{i}$. Each resultant of i iteration are combined to get convex hull.

#### Cup extraction and boundary smoothening

Canny edge detector is used to extract boundary of the detected cup region shown in Fig. 4g. Ellipse fitting is done on the boundary pixels using least square distance approach to smooth the detected surface. To represent ellipse and draw an elliptic boundary (8) is used.

8 $$x^{2} + Bxy + Cy^{2} + Dx + Ey + F = 0$$

B, C, D, E and F are the ellipse coefficients and x, y are the coordinates. Given the x and y of all the boundary pixels, ellipse coefficients are estimated such that the distance is minimized. Figure 4h shows the detected cup from the input fundus image after ellipse fitting.

### Disc segmentation

Optic disc is the pallor circular region located at the position where optic nerve leaves the eye. Optic nerve is responsible for communication between eye and brain and thus the optic disc region is a sensitive region which is also called blind spot for the reason that rods and cones (cells responsible for vision located in the internal retinal layer) are not present in this region. Optic disc detection is a fundamental and challenging step in many ocular automated disease diagnostic systems because many ophthalmic diseases are caused due to the structural changes associated with optic disc region and optic disc region is interrupted by many blood vessels. An algorithm is proposed in this section for detection of optic disc.

#### Preprocessing

**Fig. 5:**
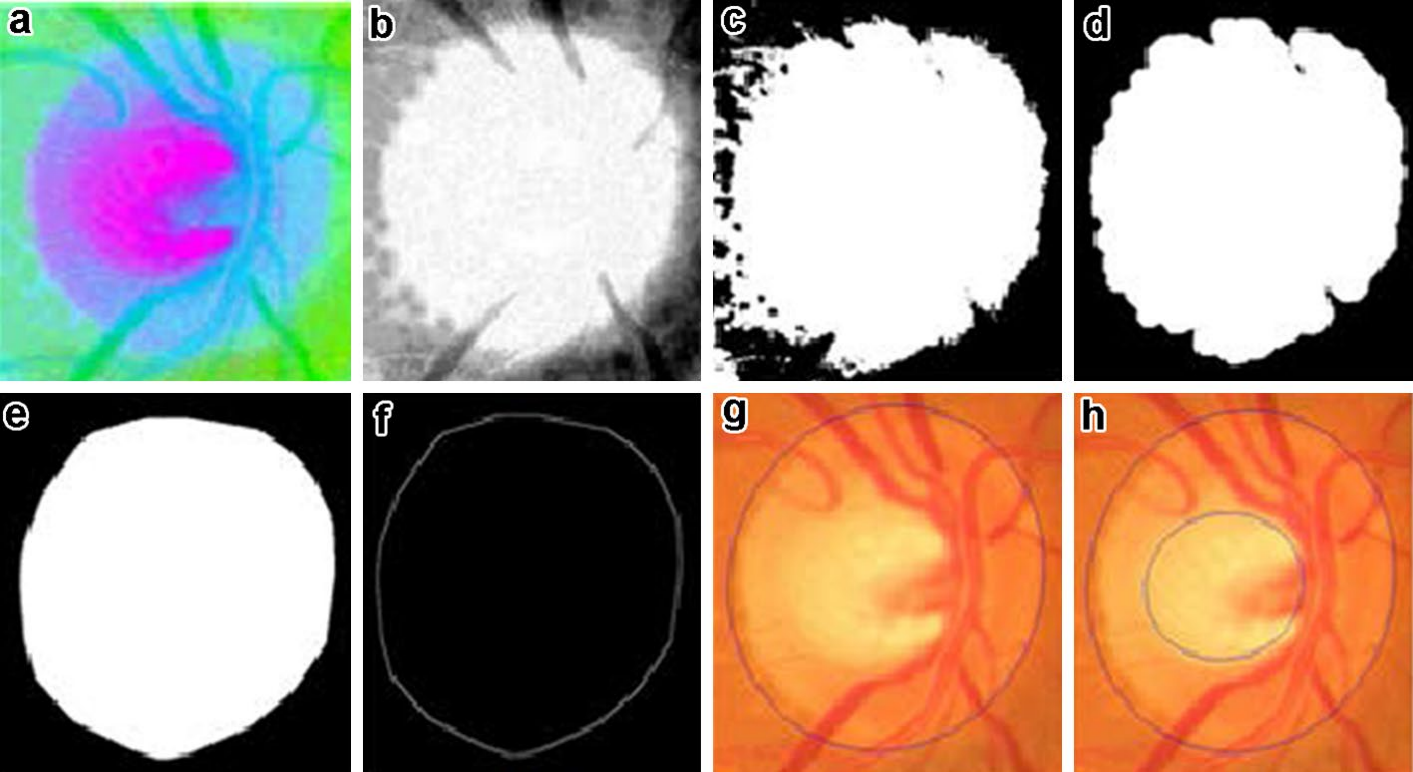
**a** Contrast enhanced image in HSV plane; **b** extracted value plane; **c** binary image; **d** results after morphological operations to remove noise; **e** convex hull; **f** edge extraction; **g** ellipse fitting and detected disc; **h** detected cup and disc

Disc detection from fundus images precedes by contrast enhancement in Hue, Saturation and Value (HSV) plane. Contrast stretching technique is used to enhance the contrast of each plane in HSV color space as shown in Fig. 5a. Optic Disc has most clear and precise impression in Value plane, thus further processing is done in value plane to extract the disc. Figure 5b shows the extracted Value plane from HSV space. Value plane is segmented into disc region and non-disc region by setting mean value of the image as a threshold. Suppose an image I {f(x. y)} where x = 1, 2, 3,…, M and y = 1, 2, 3,…, N with f (x, y) defined as pixel intensity values at x row and y column with range [0 255]. Mean of the image is calculated using (9).

9 $$\mu = \mathop \sum \limits_{y = 1}^{N} \mathop \sum \limits_{x = 1}^{M} f\left( {x,y} \right)$$

Let G {g(x, y)} be the resultant image where x = 1, 2, 3,…, M and y = 1, 2, 3,…, N with f(x, y) defined as pixel intensity values at x row and y column with range [0 1] then G will be defined as shown in (10).

10 $$g\left( {x,y} \right) = \left\{ {\begin{array}{*{20}l} {1,} \hfill & \quad {f\left( {x,y} \right) > \mu } \hfill \\ {0,} \hfill & \quad {f\left( {x,y} \right) < \mu } \hfill \\ \end{array} } \right.$$

Figure 5c shows the binary image using mean value as threshold. Resultant image G contains some unwanted noise and protrusions which are removed by performing opening operation using a disk of radius 15 as structuring element. Opening is repeated thrice and each time it is preceded by the step of removing unconnected small unwanted objects which gets isolated from the opening step as shown in Fig. 5d. The extracted disc area from the opening step may contain unfilled holes in the optic disc, originated from opening step. Convex hull of the resultant image fills the holes and unconnected parts in the optic disc area.

#### Optic disc extraction and boundary smoothening

Optic disc extracted from previous step has an irregular and rough boundary because of the opening step. To smoothen the boundary ellipse fitting is done on the boundary pixels of the ROI obtained from the previous step as shown in Fig. 5f. Boundary is extracted using canny edge detector followed by ellipse fitting on the pixels to get a smooth outline of disc. Ellipse fitting is done using the least square distance approach using the equation stated above. Figure 5g shows extracted disc after ellipse fitting on the boundary pixels obtained from Fig. 5f. Extracted cup and disc are shown in Fig. 5h.

### Feature extraction and classification

**Fig. 6:**
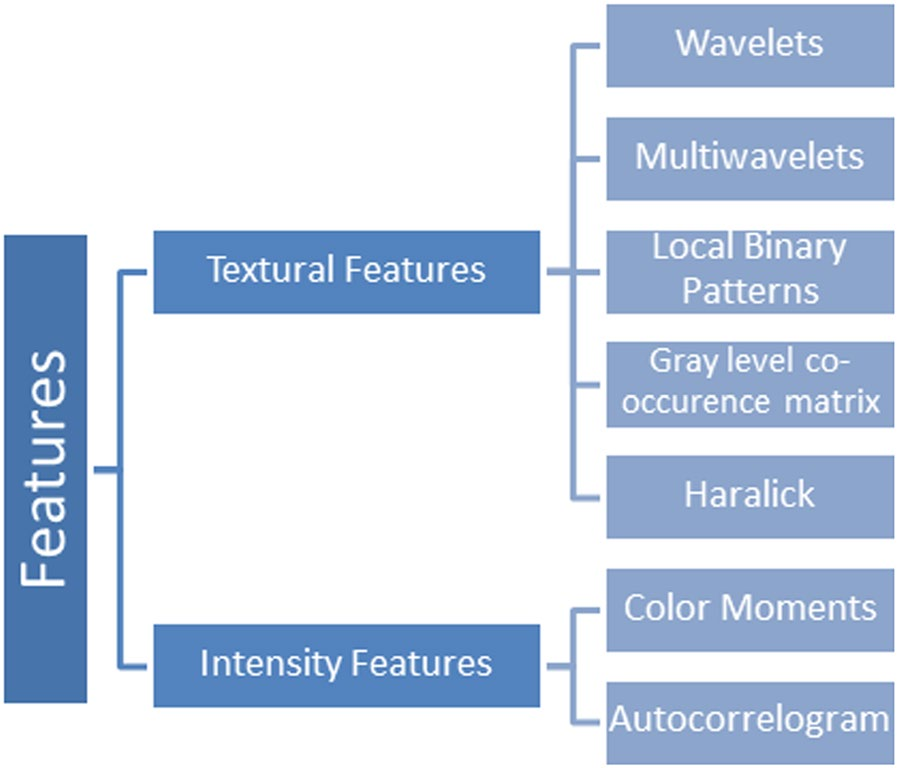
Textural and intensity based features used for glaucoma classification

Progression of glaucoma leads to an increase in size of optic cup resulting in an increase in the bright intensity pixels of the image. This increase in intensity of image can be observed in intensity and texture related features extracted from image including image entropy, histogram, mean, variance, standard deviation. Thus, in addition to the structural changes (CDR, NRR, ISNT) that are observed to detect glaucoma Intensity and texture features can also be used to classify an image as glaucoma or healthy and can be used in computer aided glaucoma systems to discriminate between glaucoma and non-glaucoma. Figure 6 shows some of the textures and intensity based features used in the proposed methodology to analyze an input fundus image and classify as glaucoma or non-glaucoma.

### Texture features

Texture is defined as specific spatial arrangement of intensities in an image. Texture features are subdivided into statistical texture features and structural texture features. In statistical texture features, pixel value’s spatial distribution is computed by calculating local features in the image. Statistical features can be first order, second order or many orders depending upon the number of pixels involved in feature extraction and computation. Local Binary Pattern (LBP) and Haralick features are statistical textural features. In structural features spatial arrangement of the pixel intensity values are computed. Wavelets, Multi Wavelets are structural textural features.

#### Wavelets

Gabor wavelet transform enable to analyze both time and frequency information at a time (Bock et al. 2007). Gabor wavelets take two inputs, a scale and number of orientations. It provides the best analytical resolution in both frequency and time domain (Forsyth and Ponce 2003). 2D Gabor function is computed using (11), (12) and (13).

11 $$\varphi \left( {x,y} \right) = \frac{{f^{2} }}{\pi \gamma n}e^{{\left( { - \left( {\frac{{f^{2} }}{{\gamma^{2} }}x_{r}^{2} + \frac{{f^{2} }}{{^{2} }}y_{r}^{2} } \right)} \right)}} e^{{2\pi fx_{r} }}$$

12 $$x_{r} = xcos \theta + ysin\theta$$

13 $$y_{r} = - xsin \theta + ycos \theta$$

Here, f is the frequency and θ is the orientation, η and $\gamma$ are self-defined constants.

#### Local binary partitions

Local binary patterns (LBP) are an approach of textural representation using neighborhood pixel intensities (Ojala et al. 2000). A comparison of enter pixel intensity (g_c_) with neighbor pixel intensities (g_0_ − g_p−1_) in an n x n neighborhood is performed. All the neighbors are separated by a distance R from the center pixel and are located at a uniform distance from each other. Coordinates of neighbor pixels are computed using (14).

14 $$\left[ {x_{p} ,y_{p} } \right] = \left[ {x_{c} + Rcos\left( {\frac{2\pi p}{P}} \right), y_{c} + Rsin\left( {\frac{2\pi p}{P}} \right) } \right]$$

Sample values of 8 Neighborhood pixels are computed with a radius 12. Difference x of center pixel intensity with the neighbor pixel is computed to get the center pixel texture information in a local neighborhood.

15 $$s\left( x \right) = \left\{ {\begin{array}{*{20}c} { 0, \quad x < 1} \\ {1, \quad x \ge 1} \\ \end{array} } \right.$$

A neighborhood weight is assigned to each pixel using the position of neighbor pixel i.e. 2^p^ if located at p position where p = 0, 1,…, P − 1. Modifying the LBP equation to compute center value becomes

16 $$LBP_{P,R} \left( {x_{c} ,y_{c} } \right) = \mathop \sum \limits_{p = 0}^{P - 1} s\left( {g_{p} - g_{c} } \right)2^{p}$$

Texture feature extraction is made uniform and rotation invariant by generating uniform, rotation invariant LBP. A pattern is uniform if transition between 1 and 0 bit occurs at max twice. To summarize the uniformity, if the difference of a generated binary code of 1’s and 0’s along with its circular shifted sequence is less than 2, pattern is said to be uniform.

17 $$U\left( {G_{p} } \right) = \left| {s\left( {g_{p - 1} - g_{c} } \right) - s\left( {g_{0} - g_{c} } \right)} \right| + \mathop \sum \limits_{p = 1}^{P - 1} \left| {s\left( {g_{p} - g_{c} } \right) - s\left( {g_{p - 1} - g_{c} } \right)} \right|$$

A more robust LBP, invariant texture feature can be extracted making the patterns rotation invariant. To achieve rotation invariant feature extraction, pattern is rotated right by 1 bit to get the minimum pattern value out of i possible patterns.

18 $$LBP_{P,R}^{ri} = \hbox{min} \left\{ {ROR\left( {LBP_{P,R} ,i} \right)} \right\} | i = 0 , \ldots, P - 1$$

Above LBP is rotation invariant and loses the directional information. Rotational invariant LBP are extracted to make the feature independent of orientations. Uniformity and rotation invariance are combined to get a uniform rotation invariant textural feature, independent of orientation and rotation.

19 $$LBP_{P,R}^{riu2} = \left\{ {\begin{array}{*{20}l} {\mathop \sum \limits_{p = 0}^{P - 1} s\left( {g_{p} - g_{c} } \right),} \hfill & \quad{U\left( G \right) \le 2} \hfill \\ {P + 1,} \hfill & \quad{U\left( G \right) > 2} \hfill \\ \end{array} } \right.$$

#### Gray level co-occurrence matrix

Gray level co-occurrence matrix (GLCM) is another statistical approach to represent textures. GLCM provides information about intensities of pixels with their relevant position thus providing spatial based structural information. GLCM for an image I of size n x n is defined in (20).

20 $$P\left( {i,j} \right) = \mathop \sum \limits_{x = 1}^{n} \mathop \sum \limits_{y = 1}^{n} \left\{ {\begin{array}{*{20}l} {1,} \hfill & \quad{I\left( {x,y} \right) = i\; and\; I\left( {x +\Delta x,y +\Delta y} \right) = j} \hfill \\ {0,} \hfill & \quad{otherwise} \hfill \\ \end{array} } \right.$$

Variables $\Delta x$ and $\Delta y$ are the distance between the pixel and its neighbor along x and y axis respectively. Intensity relationship of Neighbor pixels located at four orientations (0°, 45°, 90°, and 135°) and distance $\Delta$ are computed to calculate the GLCM, where {[0, ∆] [−∆, ∆] [−∆, 0] [−∆, −∆]} represents the displacement vector of the four orientations. GLCM for all the four orientations are computed separately and final matrix is computed by fusion of the four matrices.

#### Haralick features

Haralick features are statistical textural features computed using GLCM as basis (Simonthomas et al. 2014). Haralick features are many order features including entropy, contrast, covariance, sum of variance, difference of variance, moments. All the Haralick features used are enlisted in the table below. Where p (i, j) is defined as pixel value located at ‘i’ row and ‘j’ column in GLCM and N_g_ is the number of gray levels in the image. H_x_ and H_y_ are entropies of p_x_ and p_y_. In maximum correlation coefficient square root of second largest Eigen value of Q is computed. P_x+y_ is the probability of concurrence matrix coordinates summing to x + y.

### Intensity based features

Intensity based features are used to analyze the spatial relationship of intensities. Color based or intensity based features are the lowest level features that are rotation invariant. In Fundus images, progression in glaucoma results in an increased cup size, resulting in a change of intensity information of image due to the fact that cup region is the brightest yellowish region in the image. Thus, intensity based features can also be used to discriminate the glaucomatous and healthy fundus images. Colored and monochrome intensity based features are extracted from the fundus image to analyze the intensity relationship between pixels of an image and to classify as healthy or non-healthy image.

#### Color moments

Color moments are used to discriminate the image on the basis of intensity. Color moments are a measure of similarity of intensities between color or monochrome (gray scale) images. In color moments features, occurrence of a color in an image is represented as a probability distribution. Three color moments that are being used to analyze the color similarity of an image are mean, standard deviation and skewness. Colored image is decomposed into Red Green and Blue (RGB) plane. Each of the three moments mentioned above are calculated for each channel separately as shown in (21), (22) and (23).

21 $$\mu = \frac{1}{{N^{2} }}\mathop \sum \limits_{x = 1}^{N} \mathop \sum \limits_{y = 1}^{N} f\left( {x,y} \right)$$

22 $$\sigma = \sqrt {\frac{1}{{N^{2} }}\mathop \sum \limits_{x = 1}^{N} \mathop \sum \limits_{y = 1}^{N} \left( {f\left( {x,y} \right) - \mu } \right)^{2} }$$

23 $$S = \sqrt[3]{{\frac{1}{{N^{2} }}\mathop \sum \limits_{x = 1}^{N} \mathop \sum \limits_{y = 1}^{N} \left( {f\left( {x,y} \right) - \mu } \right)^{3} }}$$

#### Auto Correlograms

Color Correlograms are a modification of color histograms, which represents the spatial information of the pixel intensities in an image (Talib et al. 2013). Correlograms are a good representation of color information of an image using three dimensional maps. Table with entry (Ck, Cr, d) represents the probability of color Cr located at distance d from color Ck. Autocorrelogram are a modification of Color Correlograms which captures spatial information and spatial relation between pixels with identical intensity values. Local properties of an image like pixel position, gradient direction is confined to the local scope while global attributes like color distribution have a global effect. Unlike local and global parameters of an image, Correlograms encounter both the local color spatial relationship and global effects of the spatial distribution. Correlograms are extremely insensitive to the large appearance changes, unlike the other local parameters. Thus, they are good color features for intensity based classification and discrimination.

Textural and intensity based features stated above are extracted from the images to compute the feature vector. Isolated features and hybrid features (mixture of texture and intensity features) are used to train and test using two folds i.e. 50 images are used for training and other 50 are used for testing vice versa. Feature selection is done using Principal Component Analysis (PCA) to reduce the dimension of feature vector by selecting the most significant feature vectors for classification. Support vector machine (SVM) with linear kernel is used for training and testing. SVM looks for the best hyper plane that separates the two classes with the maximum distance.

## Results

### Datasets

Proposed methodology is evaluated on two local datasets. One dataset is comprised of 50 fundus images with 15 glaucoma and 35 healthy images having 415 × 452 resolution while the other is comprised of 100 fundus images containing 26 glaucoma and 74 healthy eye images having 1504 × 1000 resolution, annotated by ophthalmologists with Clinical CDR values which are used as a benchmark for the computed CDR values. Computed CDR values are compared with Clinical CDR (CCDR) values to analyze the algorithm’s accuracy. Images having CDR value >0.5 are labeled as glaucoma, whereas images with CDR value ≤0.5 are labeled as healthy eye.

### Experimental setup and results

As shown in Table 1, 24 glaucoma images are correctly detected as glaucomatous image by the proposed system, 72 non-glaucomatous images were correctly classified as non-glaucomatous images. In a nut shell, 97 images were accurately labeled by the CDR calculation algorithm, resulting in 97 % accuracy with specificity 0.98 and sensitivity 0.92. Where specificity and sensitivity are defined as

**Table 1:** Results from CDR values

	Glaucoma	Healthy
Glaucoma	24	2
Healthy	1	73

24 $$Specificity = \frac{TN}{TN + FP}$$

25 $$Sensitivity = \frac{TP}{TP + FN}$$

**Fig. 7:**
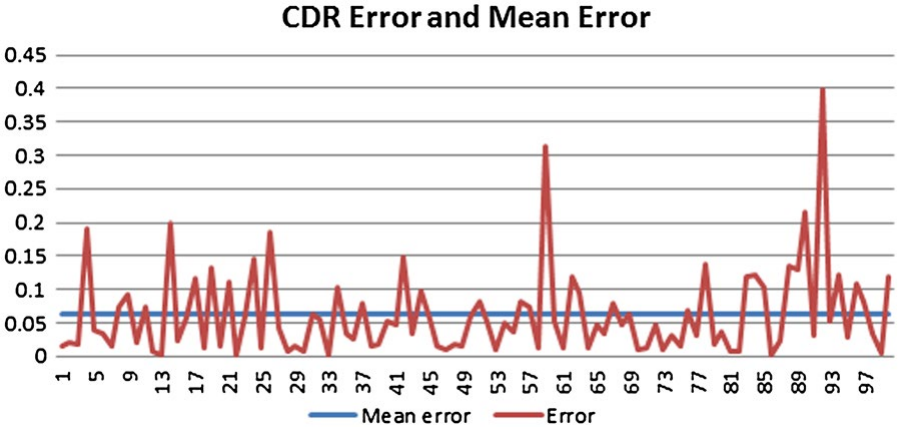
Error and mean error in CDR values compared to CCDR values

True Positives (TP) is defined as the number of samples that are glaucomatous and also detected as glaucomatous images, True Negative (TN) is defined as the samples that are healthy and detected as an healthy image, False Positives (FP) are the samples that are non-glaucomatous but detected as glaucoma and False Negatives (FN) are the samples that are glaucomatous but detected as non-glaucoma by the system. Accuracy of the system is a contribution of both Specificity and Sensitivity. Figure 7 depicts that the maximum deviation of computed CDR values from CCDR value is 0.4. Mean error of the proposed system is 0.06 and standard deviation is 0.1. Table 1 shows the image categorization results using CDR solely. Table 2 represents all the computed CDR using the proposed methodology.

**Table 2:** Results from CDR values

Image	CCDR	CDR	Image	CCDR	CDR	Image	CCDR	CDR
f1	0.3	0.32	f34	0.3	0.40	f67	0.6	0.52
f2	0.4	0.42	f35	0.4	0.43	f68	0.4	0.35
f3	0.6	0.62	f36	0.4	0.42	f69	0.7	0.64
f4	1	1	f37	0.4	0.48	f70	0.4	0.39
f5	0.4	0.36	f38	0.5	0.52	f71	0.5	0.49
f6	0.4	0.43	f39	0.4	0.38	f72	0.4	0.35
f7	0.7	0.69	f40	0.6	0.55	f73	0.4	0.39
f8	0.6	0.67	f41	0.5	0.45	f74	0.5	0.47
f9	0.3	0.39	f42	0.6	0.45	f75	0.5	0.49
f10	0.4	0.38	f43	0.5	0.47	f76	0.4	0.47
f11	0.6	0.53	f44	0.5	0.40	f77	0.7	0.67
f12	0.5	0.49	f45	0.6	0.54	f78	0.8	0.66
f13	0.3	0.30	f46	0.4	0.42	f79	0.4	0.38
f14	1	1	f47	0.6	0.61	f80	0.5	0.46
f15	0.3	0.32	f48	0.6	0.62	f81	0.4	0.39
f16	0.5	0.44	f49	0.4	0.42	f82	0.4	0.41
f17	0.5	0.38	f50	0.4	0.34	f83	0.5	0.38
f18	0.3	0.31	f51	0.5	0.42	f84	0.5	0.38
f19	0.4	0.27	f52	0.3	0.35	f85	0.5	0.40
f20	0.3	0.32	f53	0.6	0.61	f86	0.5	0.50
f21	0.5	0.39	f54	0.3	0.35	f87	0.4	0.42
f22	0.4	0.40	f55	0.4	0.44	f88	0.5	0.36
f23	0.4	0.46	f56	0.5	0.42	f89	0.5	0.37
f24	0.2	0.35	f57	0.4	0.33	f90	0.6	0.39
f25	0.6	0.59	f58	0.4	0.41	f91	0.4	0.37
f26	0.6	0.78	f59	0.9	0.59	f92	0.4	0.40
f27	0.6	0.56	f60	0.6	0.65	f93	0.6	0.55
f28	0.3	0.29	f61	0.5	0.49	f94	1	0.88
f29	0.5	0.49	f62	0.5	0.38	f95	0.4	0.37
f30	0.4	0.39	f63	0.5	0.40	f96	0.3	0.41
f31	0.4	0.34	f64	0.4	0.41	f97	0.4	0.32
f32	0.4	0.46	f65	0.4	0.45	f98	0.4	0.36
f33	0.6	0.60	f66	0.4	0.43	f99	0.6	0.60

In order to get second opinion, the proposed system uses hybrid features and SVM for detection of glaucoma. Different color, textural features are extracted and two fold training and testing (50 images for training and 50 images for testing) is done to compute the discrimination power of the extracted features. The Principal component analysis (PCA) is done for feature selection and reducing the feature matrix size by selecting the most significant features. Training and testing of all images using isolated textural and intensity based features and hybrid textural and intensity based features is done. Table 3 shows results achieved using different combinations of features. It also shows a comparison between classification accuracy of isolated features and classification accuracy of hybrid features after combining texture and intensity based features and reducing the feature matrix using PCA.

**Table 3:** Comparison Of Isolated Features And hybrid feature classification

Features	Without PCA			With PCA			
	Sensitivity	Specificity	Acc. %	PCA	Sensitivity	Specificity	Acc. %
Wavelets	0.9	0.75	86	142	0.9	0.78	87
LBP and color moments	0.87	0.9	88	15	0.88	0.9	89
Wavelets, color moments, Correlograms and Haralick	0.84	0.64	79	84	0.87	0.75	84
Wavelets and multi-wavelets	0.86	0.75	83	304	0.93	0.78	89
Multi-wavelets	0.8	0.64	76	18	0.86	0.82	85
Wavelets, color moments, Correlograms	0.84	0.57	77	3	0.87	0.68	82
Wavelets and color moments	0.88	0.75	84	77	0.9	0.78	87
Wavelets, color moments, Haralick feat.	0.84	0.75	82	192	0.88	0.82	86
Wavelets and Haralick feat.	0.82	0.75	78	185	0.83	0.86	84
LBP	0.86	0.64	80	9	0.84	0.7	81
LBP and multi-wavelets	0.86	0.75	83	18	0.86	0.82	85
LBP, wavelets, multi-wavelets	0.86	0.67	81	309	0.9	0.78	89
Haralick feat.	0.75	0.78	76	52	0.85	0.75	82
Color moments and correlograms	0.8	0.35	69	11	0.8	0.57	74
LBP, wavelets, color moments multi-wavelets	0.86	0.75	83	308	0.9	0.78	87

In case of hybrid features, maximum accuracy is achieved by combining Local binary patterns with color moments and Local Binary patterns with Wavelets and multi wavelets. However, analyzing Table 3 reveals the fact that the best combination of specificity and sensitivity i.e. highest sensitivity achieved keeping the specificity optimum is achieved using the combination of hybrid features LBP and color moments, 89 % accurate results are obtained with specificity 0.9 and sensitivity 0.88 respectively. Thus, keeps a high sensitivity rate along with an acceptable specificity. Table 4 shows the output from glaucoma detection using machine learning module. Out of 26 glaucomatous images, 2 are categorized as healthy and 24 are categorized as glaucoma. Similarly, out of 74 healthy images, 7 are falsely categorized as glaucomatous and 67 are correctly categorized as non-glaucoma.

**Table 4:** Results of machine learning module

	Glaucoma	Healthy
Glaucoma	24	2
Healthy	7	67

In autonomous medical diagnostics systems, sensitivity of a system should be close to one for the reason that all the diseased cases should be screened out. If any abnormal case is left un-diagnosed by CAD systems, it might cause an unnoticed disease progression, resulting in irreversible blindness (in case of glaucoma). In the proposed algorithm sensitivity of 0.92 is achieved by the CDR based classification, classifying one glaucoma case as non-glaucoma, sensitivity of 0.88 is achieved by feature based classification system leaving three glaucoma cases un-diagnosed. Overall system sensitivity is improved by correlating the results from both modules i.e. CDR based decision module and feature based decision module. Results are correlated in a way that the image classified as glaucoma or non-glaucoma by both the modules is labeled as diseased or healthy respectively. If results do not converge to a single decision by both modules, they are classified as suspects. Patient classified as suspect or glaucoma is directed to ophthalmologists for further examination and medications. As shown in Table 5 correlating the results from CDR and machine learning module resulted in an improved sensitivity of 1 and specificity of 0.87. Proposed methodology using a correlated result from hybrid features and structural changes gives 100 % sensitivity. Sensitivity and specificity of 1 and 0.88 respectively are observed on the dataset of 50 images. Thus the proposed system has an average sensitivity and specificity of 100 and 87 % respectively.

**Table 5:** Correlating results from CDR and machine learning

	Glaucoma	Healthy	Suspect
Glaucoma	22	0	4
Healthy	1	65	8

A technique for glaucoma detection proposed by Khan et al (2013) for glaucoma detection is used to compare the results of the proposed algorithm. Algorithm explained in Khan et al. (2013) uses CDR and ISNT rule to analyze a fundus image. A dataset of 50 images composed from different globally available fundus image datasets i.e. MESSIDOR, HEI-MED, DRIVE, STARE AND DiaRetDB0 were used for algorithm testing. Comparison of the proposed algorithm with the algorithm in Khan et al. (2013) is shown in Table 6.

**Table 6:** Comparison of results with already deployed technique

Technique	Specificity	Sensitivity	Accuracy
*Local dataset with 50 images*			
CDR based detection	0.91	0.93	92
Feature based detection	0.91	0.86	90
Combined results	0.88	1	92
*Local dataset with 100 images*			
CDR based detection	0.98	0.92	97
Feature based detection	0.90	0.88	89
Combined results	0.87	1	91
*Local dataset with 50 images*			
Glaucoma detection using CDR and ISNT rule (Khan et al. 2013)	0.85	0.73	82

## Discussion and conclusion

Glaucoma is a “silent thief of sight” having no early symptoms and can cause permanent blindness if not detected or diagnosed at an early stage. Fundoscopy enables ophthalmologists to analyze the internal retinal structural changes i.e. change in CDR, ISNT ratio. Regular retinal layer analysis is a fundamental need to prevent glaucoma or stop glaucoma progression. Research is being done in biomedical imaging to propose algorithms for CAD systems which aids doctors in analyzing and screening the affected patients. Many autonomous glaucoma detection systems help in early detection of glaucoma by analyzing the structural changes in the internal retina. CDR, ISNT ratio, NRR ratio, Vertical and horizontal Cup height are the fundamental structural changes that appear in case of glaucoma progression and are being analyzed by all the autonomous glaucoma detection systems. Many state of art Machine learning techniques are also being used in glaucoma detection systems which uses textural and intensity based features to discriminate healthy eyes from glaucomatous eyes. Proposed methodology provides an algorithm to detect glaucoma by analyzing the structural changes from fundus image and correlate the results with classification results from machine learning module. This hybrid of structural changes based evaluation and machine learning based evaluation results in more accurate results and improves the sensitivity to 1. Images labeled as healthy or non-healthy by both the modules are classified as healthy or non-healthy respectively. If the results from both modules do not converge at a decision, image is classified as suspect. Glaucoma detected cases and suspects are referred to ophthalmologists for further examination and medication. Proposed system is able to screen out glaucoma patients 100 % accuracy as none of the glaucoma case is classified as normal.
